# Surface barrier discharges for *Escherichia coli* biofilm inactivation: Modes of action and the importance of UV radiation

**DOI:** 10.1371/journal.pone.0247589

**Published:** 2021-03-17

**Authors:** Breno A. B. Salgado, Stefania Fabbri, Aaron Dickenson, Mohammad I. Hasan, James L. Walsh

**Affiliations:** Centre for Plasma Microbiology, Department of Electrical Engineering and Electronics, University of Liverpool, Liverpool, United Kingdom; VIT University, INDIA

## Abstract

Cold plasma generated in air at atmospheric pressure is an extremely effective antimicrobial agent, with proven efficacy against clinically relevant bacterial biofilms. The specific mode of bacterial inactivation is highly dependent upon the configuration of the plasma source used. In this study, the mode of microbial inactivation of a surface barrier discharge was investigated against *Escherichia coli* biofilms grown on polypropylene coupons. Different modes of exposure were considered and it was demonstrated that the long-lived reactive species created by the plasma are not solely responsible for the observed microbial inactivation. It was observed that a synergistic interaction occurs between the plasma generated long-lived reactive species and ultraviolet (UV) photons, acting to increase the antimicrobial efficacy of the approach by an order of magnitude. It is suggested that plasma generated UV is an important component for microbial inactivation when using a surface barrier discharge; however, it is not through the conventional pathway of direct DNA damage, rather through the synergistic interaction between liquid in the biofilm matrix and long-lived chemical species created by the discharge.

## Introduction

Plasma—regarded as the fourth state of matter—is a partially or completely ionised gas with unique chemical and physical properties which generates a wide range of highly reactive chemical agents when created in the ambient air. It has been increasingly studied due its antimicrobial, blood coagulation, wound healing and anti-tumour capability as well as in dentistry and several other biomedical applications [1–5]. The development of Cold Atmospheric Plasma (CAP) has brought advantages over widely used vacuum plasma technologies such as easier handling, inexpensive cost and straightforward design [6]. CAP is operated at near-room temperature and generates predominantly Reactive Oxygen and Nitrogen Species (RONS), electrons, ions and UV radiation. The specific chemical composition of CAP strongly depends on its generation parameters such as power input, frequency, voltage, working gas flow and/or composition [7].

Of the many different types of CAP reactor currently under investigation, the Dielectric Barrier Discharge (DBD) configuration is perhaps the most widely used due to its ability to create stable plasmas in molecular gases such as air. A DBD typically consists of two metallic electrodes covered by a dielectric material and separated by a discharge gap [8]. A Surface Barrier Discharge (SBD) is a subset of the DBD configuration where a stratum of plasma forms on the dielectric surface, such a configuration is inherently stable and highly amenable to being scaled over many meters [9]. One disadvantage of the SBD configuration is that short-lived RONS are confined to the active discharge region and are unlikely to reach any target placed more than a few mm downstream of the discharge layer [10]. Recent studies have focused on optimising the transport of species to a downstream target by manipulating the electrode configuration and power source [10–14]. Despite the lack of short-lived RONS, SBD’s generated in ambient air have proven highly effective for bacterial inactivation, with numerous reports demonstrating its high antimicrobial efficacy against a wide range of microorganisms grown on a variety of surfaces [4, 13, 15]. Downstream of an SBD the predominant RONS can include a mixture of O_3_ (ozone), NO (nitric Oxide), N_2_O_5_ (dinitrogen pentoxide), N_2_O (nitrous oxide), HNO_3_ (nitric acid), H_2_ (molecular hydrogen), NO_3_ (nitrate), H_2_O_2_ (hydrogen peroxide), HNO_2_ (nitrous acid) and NO_2_ (nitrogen dioxide) [16]. While many of these components have been extensively associated with bacterial inactivation via CAP [17, 18], studies with conflicting findings report the role of UV. At specific wavelengths, UV light reacts with DNA resulting in the formation of thymine dimers which impacts the bacterial cell replication capability [19]. UV is also capable of causing damage to proteins in a pattern similar to the stress caused by ROS [20] and also the bacterial membrane integrity [21]. It has been demonstrated that CAP treatment damages *Bacillus subtilis* nucleic acid majorly via UV radiation [22] and that bacterial spore inactivation by UV can be achieved after optimization of CAP operating conditions [19]. Contrarily, Dobrynin et al. [23] observed no bacterial inactivation after a plasma treatment that included a UV transparent slide positioned over the sample allowing only UV photons to pass through while preventing gaseous phase chemical species to reach the sample. Their findings agree with one of the very first studies on the role of each CAP reactive agent on bacterial inactivation, performed by Laroussi et al., where the authors concluded—based on emission spectroscopy and gas detection–that the chemical species are primarily responsible for bacterial inactivation, with minimal effects associated to UV [24].

*E*. *coli* has often been used as a model organism for studies on the bacterial inactivation efficacy of CAP. Typically, CAP is extremely effective against *E*. *coli* in both its planktonic and biofilm form. Several past studies have explored the impact of individual CAP components on inactivation of *E*. *coli* planktonic cells, including the effect of CAP generated UV on inactivation by exposing the sample to only the UV photons created by the plasma and preventing exposure from the other components created. Critically, many contrasting reports can be found and it is clear that the specific configuration and operating parameters of the CAP device play a major role in dictating the importance of UV. One study focusing on the UV isolated from an SBD discharge showed a significant effect on planktonic *E*. *coli* cells [25] whereas a study employing a plasma jet with Helium gas indicated UV radiation alone had a limited effect on *E*. *coli* monolayers on agar plates [26]. This same study also showed that a combined treatment of ROS and UV led to faster inactivation of *E*. *coli*, with indications of reactive species production by UV in the gas phase [26]. Dezest et al. [27] analysed structural modifications of *E*. *coli* suspensions exposed to CAP and although only the presence of two RONS were monitored in the plasma activated solution, the authors did not exclude the involvement of other plasma components–including UV–in the inactivation process.

In general, cells found in a sessile-biofilm mode are generally more tolerant to stress and antimicrobial challenges compared to planktonic bacteria. Previous studies have shown that CAP mediated biofilm inactivation takes longer treatment compared to planktonic cells due to the restricted diffusion of reactive species through the extracellular polymeric substance (EPS), which is known to be a major protective barrier for cell in a biofilm [28, 29]. Although the efficacy of CAP treatment on the eradication of *E*. *coli* biofilms has been demonstrated [30], little is known about the interaction between CAP bactericidal agents, biofilm cells and the EPS.

This study aimed to uncover the modes of bacterial biofilm inactivation mediated by an SBD operating in ambient air. It was hypothesised that the antibiofilm effect exhibited by CAP arises though a synergistic interaction between the plasma generated RONS, UV photons and the biofilm matrix. To determine the relative importance of CAP generated antimicrobial agent as well as identify synergistic interactions occurring, a variety of different SBD reactor configurations were adopted and their inactivation kinetics examined alongside experimental measurements and computational predictions. Our results demonstrate that effective microbial inactivation is significantly enhanced through the synergistic interaction between CAP generated UV photons and long-lived RONS when they interact with the EPS matrix of the biofilm.

## Material and methods

### Bacterial strains and culture conditions

The *E*. *coli* strain considered in this study, namely BW25113, is the parent strain of the Keio collection and was acquired from Horizon Discovery Ltd (Cambridge, United Kingdom) and stored at -80°C in Luria Broth (LB, Sigma-Aldrich Company Ltd, Gillingham, United Kingdom) supplemented with 20% (v/v) glycerol (Sigma-Aldrich). Bacterial cultures were prepared from a single colony inoculated into 10 ml of broth LB medium and incubated overnight at 37°C at 160 rpm in a shaking incubator (SI500; Stuart Equipment, Staffordshire, United Kingdom).

### Biofilm formation

Overnight *E*. *coli* cultures were adjusted with fresh LB broth medium to a final concentration of 1 x 10^7^ CFU/ml. Biofilms were generated on polypropylene CDC coupons (Bio Surface Technology Corp., Bozeman, MT, USA). Each coupon was placed into a well of a 24-well plate prior to the addition of 1 ml of fresh *E*. *coli* culture. Sterilization controls of LB broth and coupons were included for every used 24-well plate. Biofilms were grown for 24 h in static conditions.

### Plasma system configuration

**Fig 1:**
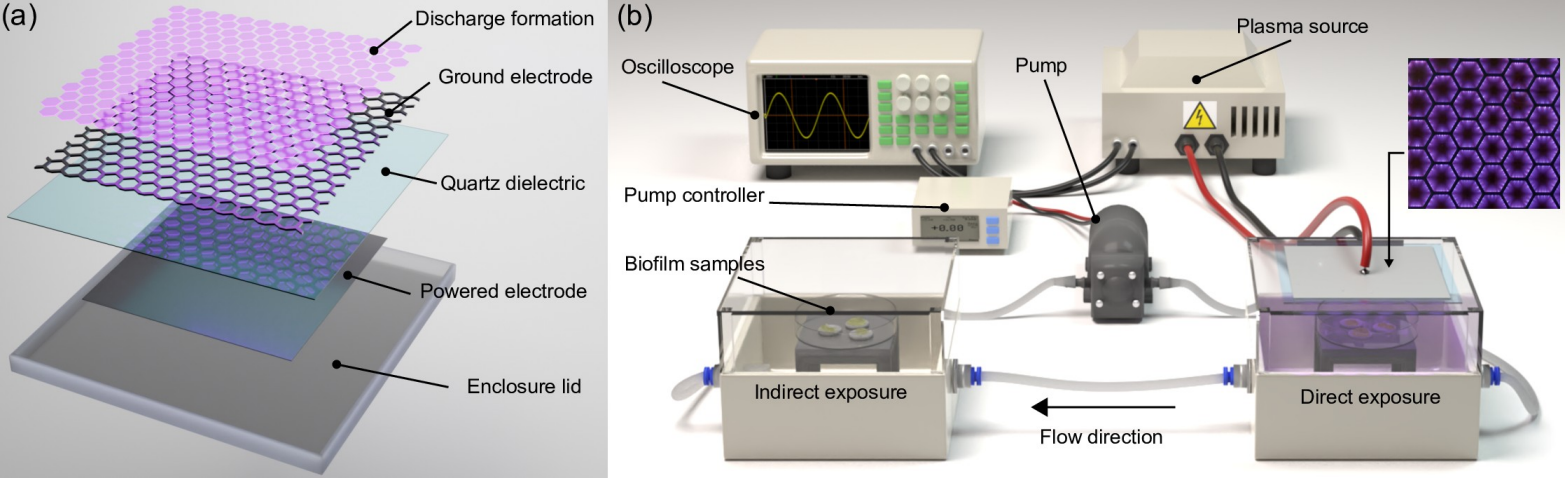
Diagrams showing configuration of: (a) the Surface Barrier Discharge for plasma generation, and (b) the direct and indirect biofilm treatment enclosures alongside the power source and gas pumping system.

The SBD used in this study consisted of a grounded mesh stainless steel electrode containing hexagonal gaps in which the plasma discharges were formed. The mesh electrode was separated from a high voltage copper plate electrode by a 2 mm thick, 5 cm^2^ quartz plate, acting as the dielectric barrier, as shown in Fig 1A. The high voltage (HV) electrode was shrouded with Kapton tap in order to inhibit the formation of plasma along its edges. The SBD was attached to the upper inner surface of a 110x80x85 mm enclosure, to allow the reactive species formed by the plasma generating electrode to diffuse downwards onto the biofilm containing coupons.

A 19 kHz HV signal applied to the powered electrode was sourced by a home-made power inverter. This inverter was fed by a DC voltage supply (GW Instek PSP-603) and driven by an AIM-TTI TG2000 signal generator. The experiments were performed at a discharge power condition of 1.65 W.cm^-2^. The electrical characteristics of the HV signal were monitored using a Tektronix HVP-15HF voltage probe, Pearson 2877 current monitor and Teledyne LeCroy T3DSO1000 oscilloscope. Throughout each experimental run, the power dissipated in the plasma was calculated in real-time and held at a constant level.

### Plasma treatment

To treat biofilm coated-coupons with CAP the configuration shown in Fig 1B was employed. The setup allowed for samples to be placed in either the ‘direct’ enclosure, where they would be positioned close to the CAP generating electrode and thus be subjected to both RONS and UV; or the ‘indirect’ enclosure where they would only be subjected to longer-lived RONS. A small air pump was used to draw gas from the ‘direct’ enclosure into the ‘indirect’ enclosure and a return line was fitted to ensure a closed system.

Samples in the direct treatment enclosure were treated 3 mm away from the SBD electrode, while samples in the indirect enclosure were positioned approximately in the centre of the enclosure. Samples from both enclosures were treated for 60, 120 and 180 s on both sides of the coupon. Similar to previous studies on biofilm inactivation using SBD-based devices, the dissipated power within the discharge was held constant at a value that rapidly led to ozone poisoning; consequently, biofilms were primarily exposed to a RNS dominated gas phase chemistries. Such conditions are known to be favourable for efficient inactivation [4]. All tests were conducted in triplicate.

For tests where plasma generated UV was to be prevented from reaching the biofilm samples, a quartz disc was sputter coated with copper using a Q150T ES turbomolecular-pump coating system (Quorum Technologies Ltd, East Sussex, United Kingdom). The coated disc was confirmed to completely block all wavelengths that were expected to be produced by the plasma. During the tests the coated disc was placed between the SBD electrode and coupons to block UV transmittance from the SBD to the sample.

### Nitric oxide assay

To assess the impact of nitric oxide on biofilm inactivation an enclosure similar to that used for plasma treatments was adopted. A mass flow controller connected to a pre-mixed gas cylinder containing 1000 ppm of NO in N_2_ (BOC, Manchester, United Kingdom) was used to control the flow of gas in the treatment chamber. A second flow controller connected to a nitrogen gas cylinder was used to enable dilution of the gas within the treatment chamber, enabling NO concentrations of 10–1000 ppm to be obtained. The chamber was vented through a small hole into a gas filtration and extraction unit. Biofilm coupons were placed in the enclosure and exposed to NO mixtures at a constant flow of 1 L/min for 1, 2, 3, 10 and 15 min on both sides.

### Determination of biofilm inactivation

Bacterial survival following plasma and nitric oxide exposure was determined by serial dilution with the Miles–Misra method as used by Modic et al. with minor modifications [4]. Briefly, the control and treated polypropylene coupons were immediately transferred to 15 ml centrifuge tubes (Appleton Woods Ltd, Birmingham, UK) containing 5 mL of LB broth and then vigorously shaken for 15 min using a vibratory shaker (VXR basic Vibrax®; IKA, Staufen, Germany) at 1800 rpm to dislodge and disrupt biofilm cells, releasing them into the media. Further, 20 μL of the bacterial suspension was mixed with 180 μL of fresh LB broth using Corning® Costar® 96-well polystyrene plates (Corning Life Sciences, Tewksbury, MA). This was serially diluted 10-fold to 10^−4^ and triplicates of 10 μL for each dilution from controls and exposed samples were plated on LB plates. Plates were incubated overnight at 37°C until visible colonies could be counted.

### Optical emission spectroscopy

Optical emission spectroscopy was employed to gain an appreciation for the excited states within the plasma. An Andor Shamrock SR-500i-A spectrometer with an Andor iStar iCCD DH734-18U-03 detector were used to capture the spectrum from the SBD and that of an Ocean Optics DH2000 lamp using both the Deuterium and Halogen lamps for the purpose of irradiance calibration. A 50 μm entrance slit was used with a 1 ms exposure, with the detector cooled to -10°C. Each spectra shown is comprised from 10 individual acquisitions. The spectrometer was calibrated to give the absolute intensity of the discharge (μW.cm^-2^.nm^-1^) using data provided by the DH2000 lamp manufacturer and the diameter of the collecting fibre.

### FTIR spectroscopy

Fourier Transform Infrared Spectroscopy (FTIR) spectroscopy was employed in order to analyse the composition of various long-lived plasma generated species. A Jasco FT/IR-4600 spectrometer was used for the measurements of the IR spectra from 600–3000 cm^-1^, with an acquisition resolution of 8 cm^-1^. Spectral acquisitions were taken every 30 seconds after plasma ignition for a total period of 180 seconds, matching the total treatment times used in the biofilm decontamination experimentations. Each dataset is presented as the average of 30 individual acquisitions. During each measurement, an air pump was used to flush the long-lived species out of the direct treatment chamber and into a gas cell (with a path length of 10cm) placed in the FTIR spectrometer for analysis. The gas cell was fitted with potassium bromide (KBr) windows to transmit the full range of the IR spectra under investigation through the cell. Absorption measurements were conducted for the discharge operating at 1.65 W.cm^-2^.

### Computational modelling

The numerical model used in the study is identical to that reported by Hasan & Walsh [31]. Briefly, a coupled set of 1D convection-diffusion-reaction equations is solved, in space and time, for the mass fractions of 53 species. These include electrons, positive and negative ions, excited state neutral species and neutral reactive species, interacting through 624 reactions. A complete list of the species and reactions considered was reported by Sakiyama and co-workers [32]. The background air is assumed to consist of 97% N_2_, 20% O_2_ and 1% H_2_O. It was assumed that the discharge is driven by a Gaussian pulse of electric field with a duration of few nanoseconds, mimicking the lifetime of a filamentary discharge; this field is converted to a mean electron energy through the local field approximation [32]. To mimic the experimental conditions a convective flow of 1 L/min was introduced into the model.

**Fig 2:**
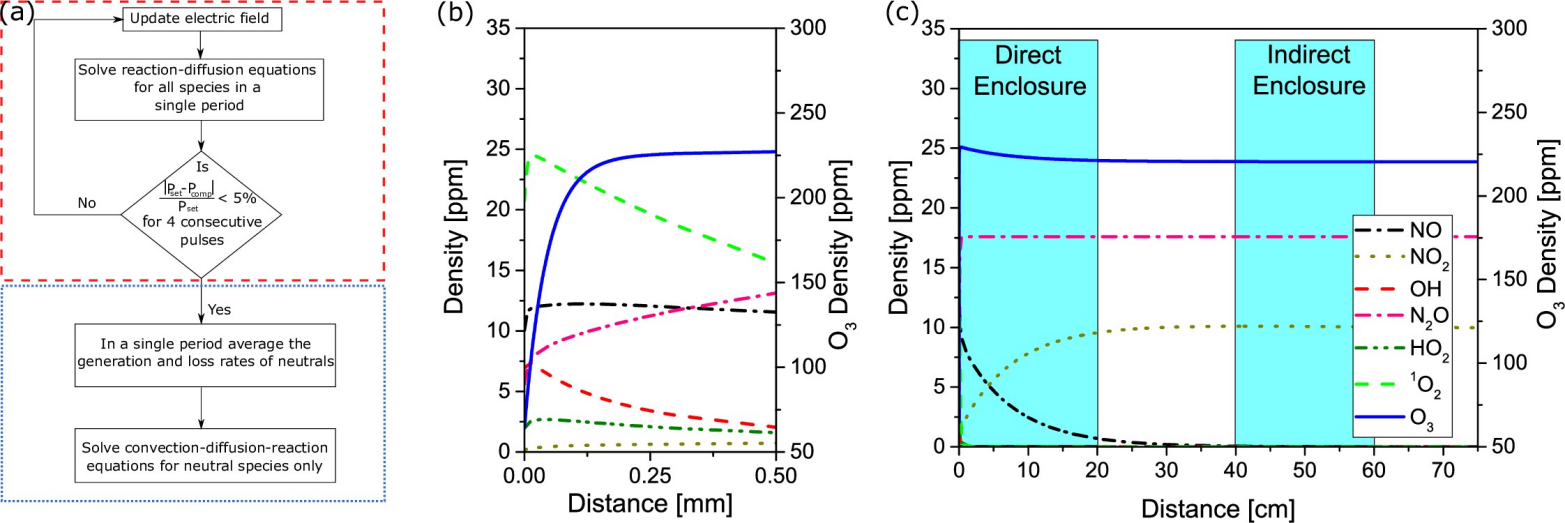
Computational approach to assess lifetime of key RONS. (a) flow-chart showing modelling approach to assess transport of long-lived reactive species from plasma region, (b) and (c) show calculated species densities as a function of distance downstream of the plasma generating electrode, with (b) emphasising the rapid drop in short-lived species densities close to the SBD electrode and (c) emphasising the downstream propagation of long-lived species between the direct and indirect enclosures.

To calculate the density of reactive species as a function of distance away from the plasma generating electrode, the computational procedure shown in Fig 2A was followed. In the first step, the model was solved for all species, assuming the discharge is ignited in the first 40 μm of the domain, representing the plasma region of the discharge. At the end of each period of the applied waveform the average power was calculated and the magnitude of the electric field modified to adjust the average power in the next period, to match that of the experiment. This procedure was repeated until the average power computed differed by less than 5% from that of experiments. If five consecutive periods of the applied waveform satisfied this condition, the generation and loss rates of neutral species were averaged over the last period of the waveform. In the second step the model is solved only for the long-lived species for 10 seconds, taking into account the time-averaged generation and loss rates obtained from the first step. This procedure allows for the effect of short-lived species, such as electrons and ions, to influence the chemistry of the neutral species without needing to resolve every period of the applied waveform.

## Results

### Computed species profiles

The distribution of plasma generated species as a function of distance from the plasma generating electrode is shown in Fig 2B and 2C, a clear difference in the gas phase RONS composition can be observed depending on distance from the plasma generating electrode. In close proximity to the electrode, Fig 2B, highly oxidizing short-lived species, such as OH, are predicted. As distance from the electrode increases, the density of short-lived species rapidly drops below the ppm level and therefore, unless samples are placed extremely close to the electrode which is typically not practical, the direct role of these species in microbial inactivation is likely to be minimal. Notably, only four species have a concentration above 1 ppm at distances >1 mm downstream of the electrode; these include O_3_, N_2_O, NO_2_ and NO. As shown in Fig 2C, of these four species, only NO shows an appreciable drop in concentration with distance from the electrode. Consequently, biofilm samples placed in the direct chamber are likely to be exposed to all four species, while those in the indirect enclosure, located approximately 50 cm away from the SBD electrode, are only likely to be exposed to N_2_O, NO_2_ and O_3_.

### Plasma and gas phase characteristics

**Fig 3:**
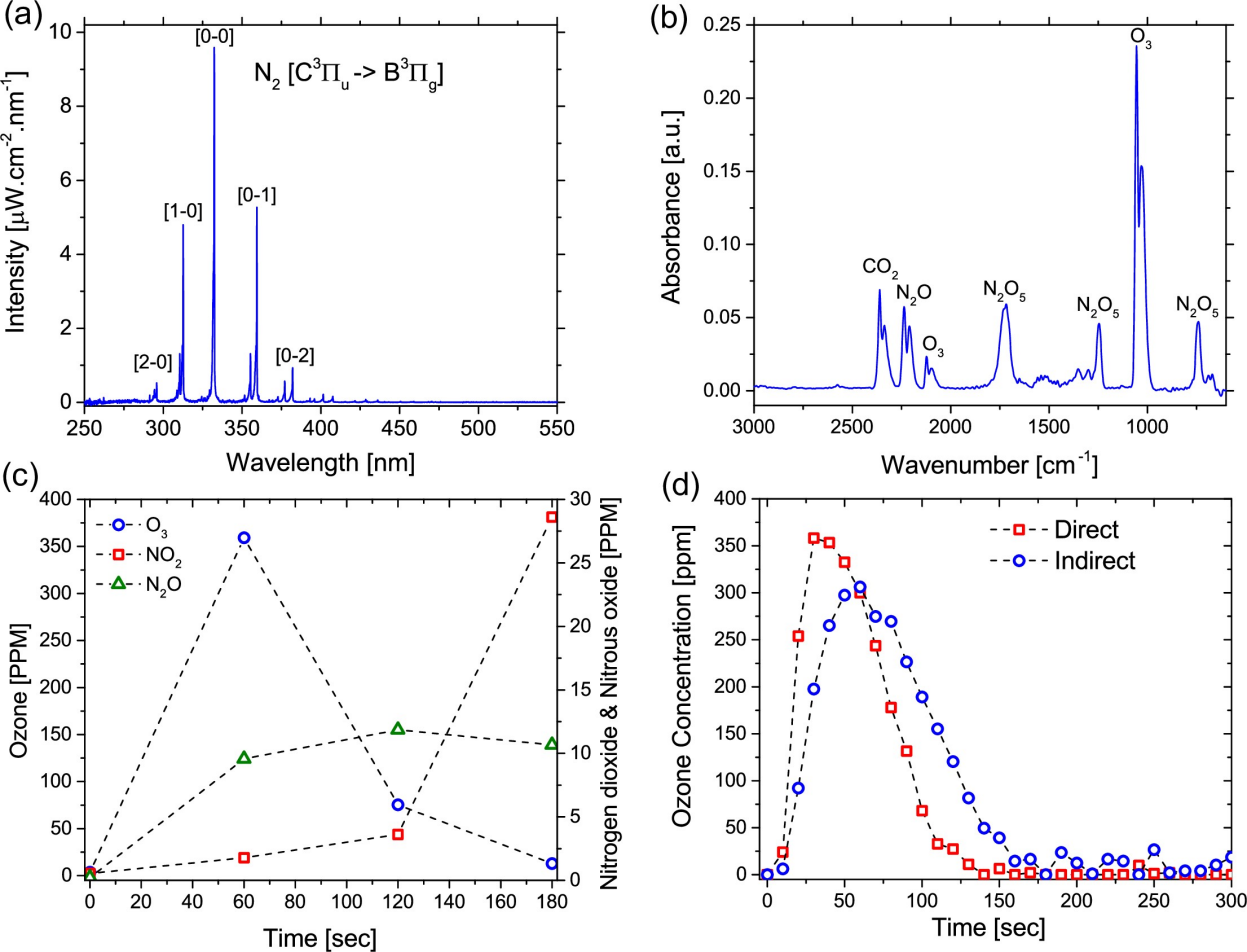
Results from plasma characterization activities. (a) Absolute emission intensity from the SBD electrode measured using optical emission spectroscopy, (b) Typical FTIR absorption spectra showing characteristic peaks for O_3_ and various NO_x_ species, (c) Time evolution of O_3_, NO_2_ and N_2_O at a plasma power of 1.65 W.cm^-2^, and (d) Simultaneous measurement of Ozone density in both the direct and indirect treatment reactor.

Optical emission spectroscopy was used to gain an appreciation for the excited states within the discharge and the intensity of light emitted from the plasma. Fig 3A shows the emission spectra from 250 to 550 nm. It is worth noting that no emission above 550 nm was detected up to the upper wavelength limit of the detector (~ 850 nm). As it is typical for a low temperature discharge in air at ambient pressure, the emission spectrum was dominated by the nitrogen second positive band system. The emission from the system extends from 290 nm to 450 nm, with a peak at 337 nm. Notably, the emission from the discharge spans the UVA (315–400 nm) and UVB (280–315 nm) region, it does not extend into the germicidal UVC portion of the spectrum (180–280 nm). Furthermore, a maximum intensity of 9.5 μW.cm^-2^ was measured at 337 nm, a value that is approximately 19 times lower than that measured from a typical 5 W germicidal lamp operating at 254 nm [33].

As shown in the modelling results, beyond the discharge layer short-lived RONS rapidly react to form longer-lived products, many of these species can be quantified using FTIR. The evolution of several RONS in the gas phase was assessed using FTIR spectroscopy. Fig 3B shows the absorption spectra of the discharge effluent after 60 seconds of plasma generation. It is clear that significant concentrations of both reactive oxygen and nitrogen species such as O_3_, N_2_O_5_ and N_2_O were detected within the FTIR gas cell. Fig 3C shows the time evolution of IR active RONS recirculating through the system. After 60 seconds of plasma generation, ozone poisoning was seen to occur, observed as a reduction in the ozone absorption peaks at around 1025 and 2100 cm^-1^. After 180 seconds absorption due to ozone was found to be completely inhibited. A shift in the composition of the gas phase chemistry was observed and nitrogen oxides were found to dominate. An absorption peak around 1600 cm^-1^ developed indicating the initiation of NO_2_ generation in the gas phase. Such a transition is often observed in air fed SBD devices and is attributed to the reaction of NO with O_3_ which leads to the formation of NO_2_ and diminishes O_3_ concentration [34]. Absorption by N_2_O_5_ was also observed to reduce after this transition point, as seen by the reduced absorption peaks around 740, 1250 and 1720 cm^-1^ after 120 seconds. Fig 3D shows the evolution of ozone density within the direct and indirect enclosures during plasma generation. The results indicate that the concentration of ozone in the indirect chamber lags behind that of the direct chamber. This is to be expected as transport of ozone over tens of centimetres into the indirect chamber will take several seconds at the investigated flow rate. It is also clear that, while the concentration profiles are qualitatively similar in both enclosures, quantitatively the peak concentration was approximately 15% lower in the indirect treatment chamber. This difference is a likely consequence of the loss processes occurring, such as the reaction between O_3_ and NO, as described in our previous works [35].

### Effect of direct and indirect plasma treatment on *E*. *coli* biofilms

**Fig 4:**
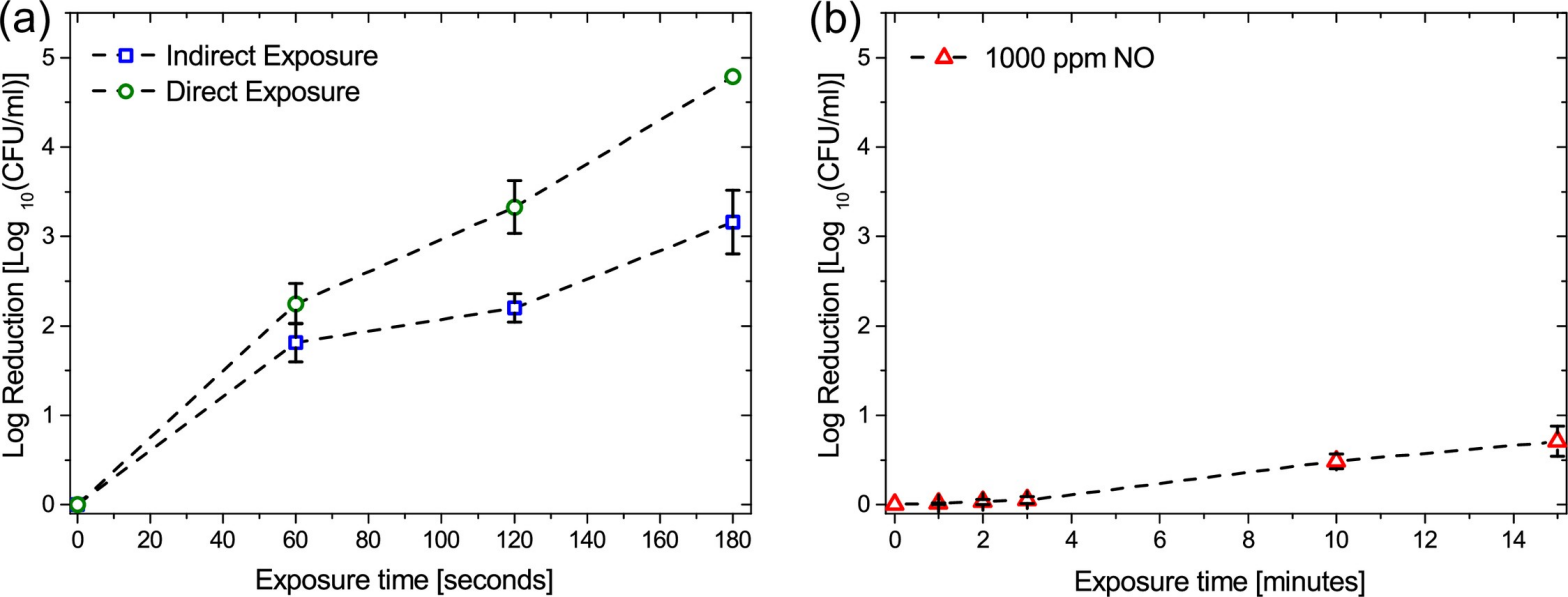
Inactivation of *E*. *coli* biofilms. (a) Impact of exposure time on microbial inactivation in the direct and indirect treatment reactors, and (b) Impact of exposure time on microbial inactivation of 1000 ppm NO premixed in N_2_.

Biofilms of *E*. *coli* BW25113 grown on polypropylene coupons were exposed to direct and indirect CAP treatment using the reactor configuration shown in Fig 1B and microbial inactivation was evaluated after 60, 120 and 180 seconds of exposure. As indicated in Fig 4A, direct treatment shows an overall higher efficiency in the biofilm inactivation rate, with no bacterial cells detected after a 180 second exposure. During the first 60 seconds of exposure both direct and indirect treatment eliminated biofilm cells similarly. while a 120 second exposure showed increased eradication only for the biofilm directly exposed to CAP. A 180 second exposure was found to eliminate all bacterial cells within the biofilm only under direct treatment, reaching the 2x10^2^ CFU limit of detection for the test. Conversely, a reduction in the rate of inactivation was observed for indirectly treated biofilms beyond 60 seconds of exposure.

This finding clearly indicates that biofilms placed within the direct enclosure experienced different treatment conditions to those in the indirect enclosure. However, such a finding contradicts the results of the gas phase characterization presented in Figs 2 and 3, which indicate that a biofilm placed ~ 1 cm from the discharge would receive an approximately similar ‘dose’ of reactive species as a biofilm placed ~ 50 cm away from the discharge. The only reactive agents that do not reach the indirect enclosure are UV photons and NO, which is readily lost through reaction with ozone. Consequently, these findings indicate that either UV, NO or both play a significant role in the antimicrobial action of an SBD.

### Effect of NO on *E*. *coli* biofilm

The biological importance of NO is well established and it is known to have powerful antibiofilm properties. Based on computational modelling (Fig 2) and previous experimental studies of NO production and transport in an SBD [10], it has been determined that NO concentrations can reach several hundreds of ppm under operating conditions similar to those used in this study. To investigate the antimicrobial efficacy of NO against of *E*. *coli*, biofilm coated coupons were placed in an enclosure containing various concentrations of NO ranging from 10 ppm to 1000 ppm for up to 15 minutes. Fig 4B shows only the case of 1000 ppm NO, as lower concentrations were found to be ineffective for microbial inactivation. Following exposure to 1000 ppm of NO premixed in N_2_ gas, a slight bacterial reduction was apparent only after 10 minutes of treatment, with a 0.5 log reduction observed after 15 minutes. It is worth noting that such NO concentrations greatly exceed those that are likely to be experienced during plasma treatments, thus these findings suggested that NO alone is not solely responsible for the differences observed in microbial inactivation between the direct and indirect plasma exposure cases.

### Contribution of plasma generated UV on *E*. *coli* biofilm inactivation

**Fig 5:**
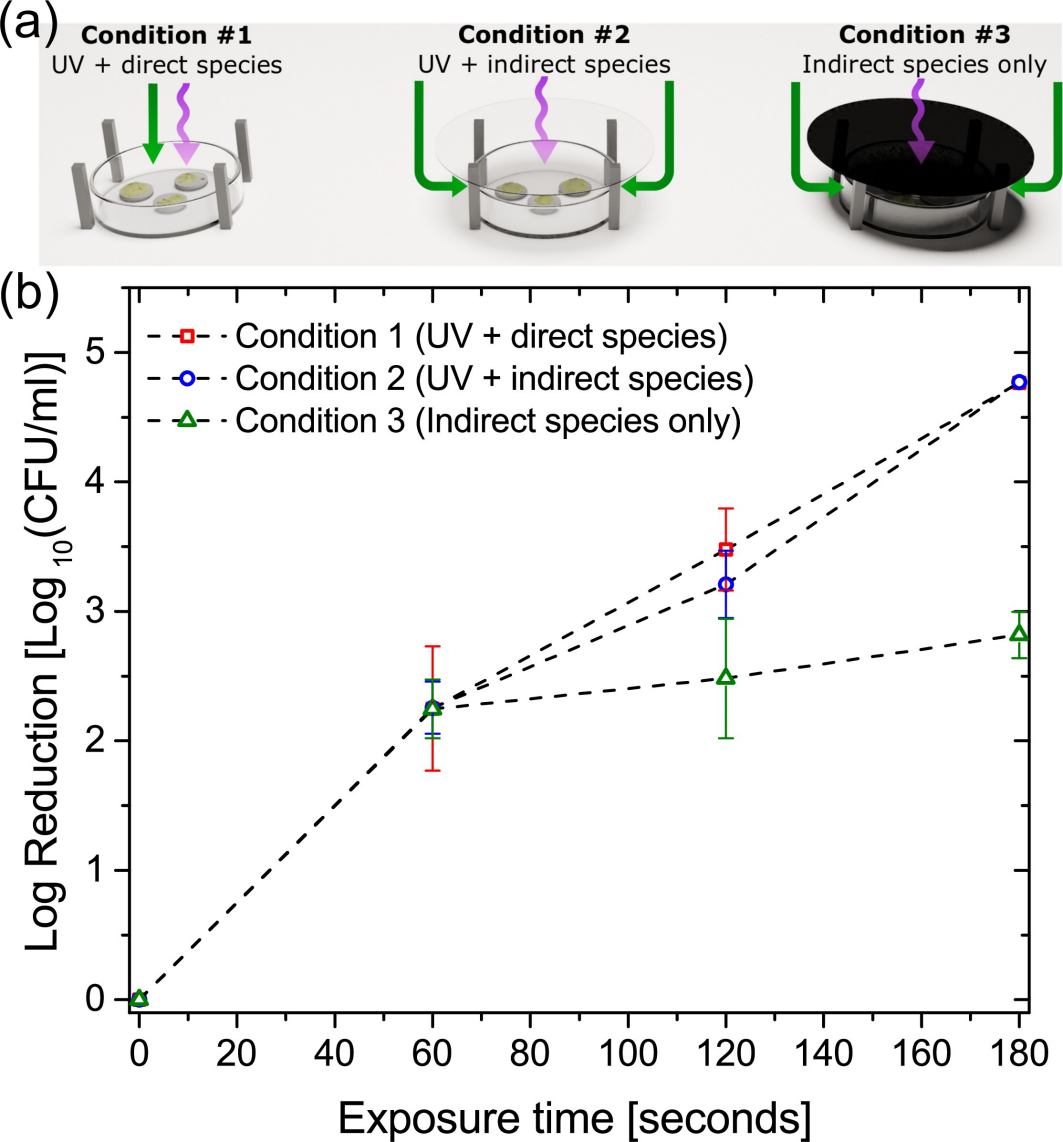
Modes of action and the exclusion of EHD driven mass transport effects. (a) experimental configuration showing three treatment conditions: (i) biofilms exposed to both UV and chemical species transported from the plasma, (ii) quartz blocking plate used to allow transmission of UV while preventing direct transport of species from plasma, and (iii) Opaque blocking plate preventing transmission of UV and direct transport of species from plasma. Part (b) shows biofilm inactivation results for the three conditions tested.

To assess the contribution of plasma generated UV towards the inactivation of *E*. *coli* biofilms a modification was made to the direct treatment enclosure whereby a quartz disc (UQG Ltd, Cambridge, UK) was placed between the SBD electrode and biofilm samples, Fig 5A. Three specific conditions were investigated: (i) no quartz disc–where samples were exposed to both the reactive species and UV from the discharge; (ii) UV transparent quartz disc–where samples were exposed to UV from the discharge and any reactive species that were able to diffuse around the disc; and (iii) quartz disc sputter coated with copper to block UV transmittance–where samples were exposed to only the reactive species that were able to diffuse around the UV blocking disc.

Fig 5B shows the inactivation results from the three cases examined. UV-treated biofilms conditions (i) and (ii) showed higher susceptibility in comparison with the UV-untreated biofilms. UV-treated biofilms were reduced to levels below the detection limit of the assay whereas UV-untreated biofilms showed a 2-log survival after 180 seconds of exposure. It is clear that conditions (i) and (ii) give very similar inactivation results, which is perhaps expected as biofilm samples under both treatment conditions were exposed to both UV and reactive species; however, it is well known that the dominant transport mechanism of reactive species in an SBD is convection resulting from electrohydrodynamic (EHD) forces created by the plasma [35]. Convective flows of ~ 1 m/s are typical for SBD’s operated under conditions similar to those examined in this investigation. It is well documented that such flows can potentially enhance the transport of short-lived species from the discharge region to a downstream sample and thus enhance the apparent antimicrobial efficacy of the discharge [13]. With no quartz disc used in condition (i) the convective flow of species from the discharge impinge directly on to the biofilm samples; conversely, the window used in condition (ii) blocks gas flows originating from the discharge and thus species transport to the sample is limited to diffusion only. Consequently, comparing the inactivation efficacy between conditions (i) and (ii) demonstrates that EHD forces have little impact on the antimicrobial efficacy of the discharge under the conditions examined in this investigation.

**Fig 6:**
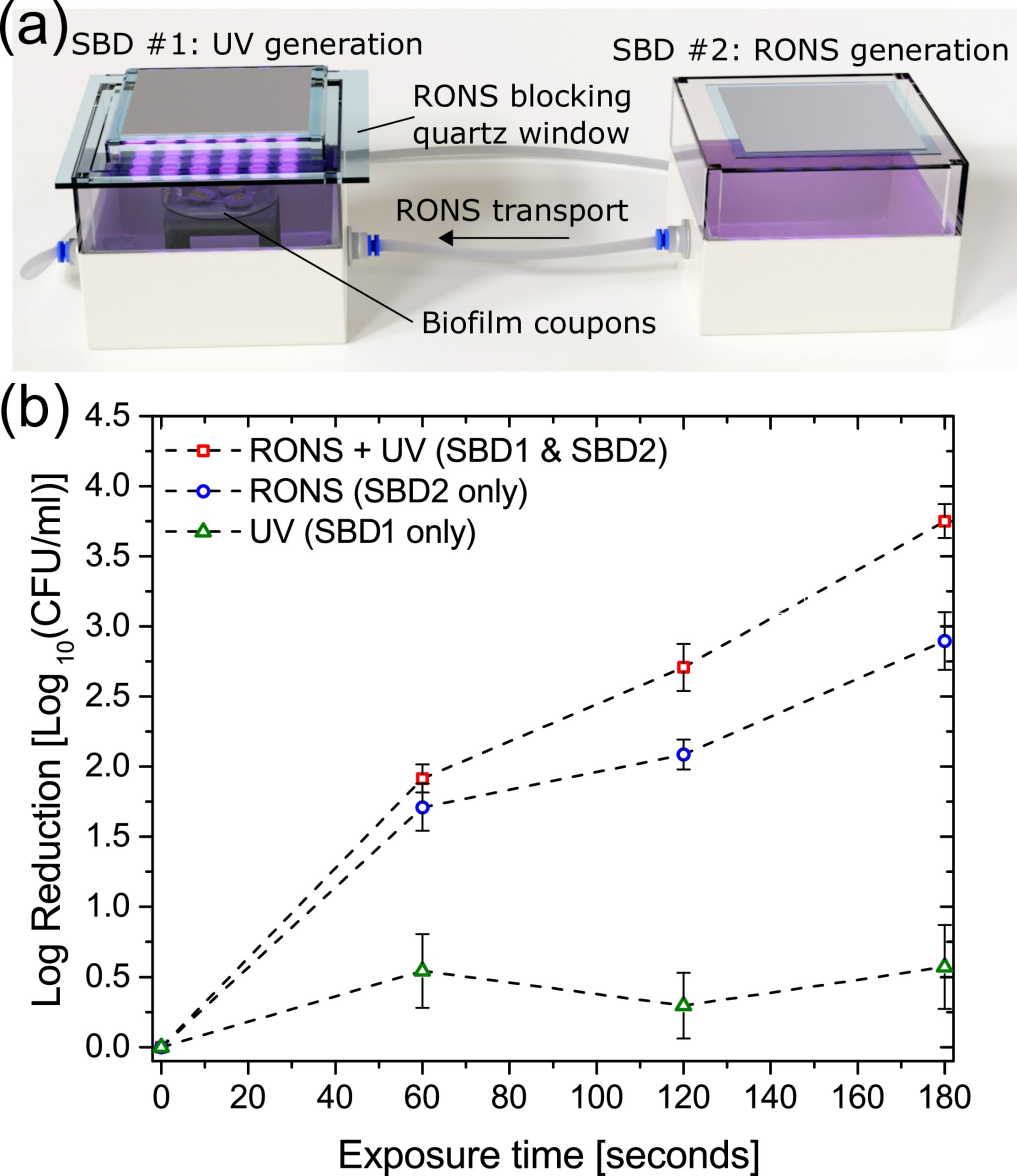
Synergistic effects of UV illumination. (a) experimental configuration involving two identical SBD’s to simultaneously generate long-lived species and UV, and (b) Inactivation results showing three test cases: (i) Long-lived RONS and UV from external source, (ii) Long-lived RONS only and (iii) UV from external source only.

Finally, in order to examine for possible synergistic effects between UV, long-lived RONS and the biofilm samples, a further modification was made to the experimental setup, as shown in Fig 6A. A second identical SBD was mounted above the indirect treatment enclosure and a quartz window was used to block all species transport into the enclosure, allowing only the transmission of light from the discharge to the biofilm samples within the enclosure. Using such a setup enabled the interaction between plasma generated UV and long-lived species created and flushed from within the ‘direct’ exposure enclosure to be investigated.

Coupons-containing biofilms were treated for up to 180 seconds with either (i) long-lived reactive species pumped from the ‘direct treatment’ enclosure with external UV illumination, (ii) only the long-lived reactive species pumped from the ‘direct treatment’ enclosure, and (iii) UV illumination from the external SBD (*i*.*e*. no chemical species) (Fig 6B). The CFU count showed that UV exposure alone had very little impact on bacterial cells within the biofilm, which was not unexpected given the comparatively low intensity of UV emitted from the plasma source, shown in Fig 3A. Conversely, biofilms treated with both UV and long-lived RONS were more efficiently reduced compared to biofilms treated with only the long-lived reactive species. In line with the bacterial reduction observed in Fig 5, it is clear that UV plays an important, albeit indirect, role in biofilm inactivation mediated by a SBD. The reduction observed in this approach was similar to that found for a direct exposure, shown in Fig 4A, demonstrating that a synergistic interaction between UV and long-lived species generated by the plasma is required for rapid and effective microbial inactivation.

## Discussion

This study demonstrated the inactivation mechanisms of a SBD-CAP system on *E*. *coli* biofilms. Direct and indirect treatments both successfully reduced viable counts of *E*. *coli* biofilm cells corroborating previous studies that show efficient application of plasma in inactivation of *E*. *coli* [27, 36–38]. Successful inactivation of *E*. *coli* via plasma exposure has been reported for a variety of plasma operating conditions including frequency, voltage and working gas [30, 39]. It is well known that CAP generates a wide range of RONS with bactericidal effect, including atomic oxygen, superoxide, ozone, hydrogen peroxide, hydroxyl radicals and nitric oxide [40, 41]. In the specific case of a SBD, many of these short-lived RONS are not transported beyond the visible plasma region and are thus confined to within <<1 mm of the plasma generating electrode and cannot play a direct role in the inactivation of biofilms situated >> 1 mm away.

In this study, a comparison of indirectly and directly exposed samples showed clear differential biofilm reduction kinetics. Samples exposed to a direct treatment experienced higher and complete inactivation compared to indirectly treated samples. In both cases, the biofilm samples are situated >> 1mm away from the discharge layer and thus are likely to receive a similar flux of reactive chemical species, as demonstrated by the plasma characterization results shown in Fig 3. This is in agreement with the simulation results presented in Fig 2, and previous works detailing the numerical simulation of the SBD-CAP system; where it was demonstrated that a maximum of four reactive chemical species are able to travel further than 1 mm away from the discharge region [32]. The only exception to this is NO, which is considered to have an ‘intermediate’ lifetime in the effluent of an SBD. NO readily reacts with O_3_ to form NO_2_, thus its concentration drops beyond the discharge layer, as shown in Fig 2C. Given this, it was anticipated that samples situated in the direct treatment enclosure would receive a significantly higher dose of NO compared to those in the indirect enclosure, thus provoking the hypothesis that NO may be a key factor in dictating the antimicrobial efficacy of the SBD.

NO is a small gas molecule with one unpaired electron which is produced by host cells during *E*. *coli* infection and has antibacterial activity, including multidrug-resistant isolates [41, 42]. Due its ability to easily diffuse through biological membranes, it has been suggested that NO is a suitable agent against Uropathogenic *Escherichia coli* (UPEC) trapped within biofilm communities or host cells [43]. Although micromolar concentrations of NO are toxic to bacteria [44], our results revealed that the NO concentration required for *E*. *coli* inactivation was substantially higher than the NO density likely to be found in the direct treatment enclosure. Critically, the concentration of NO used in our experiments (1000 ppm) was approximately ten times higher than what was measured from the discharge in our previous works [35]; furthermore, lower concentrations including a more realistic 10 and 100 ppm were found to have no impact on the biofilm samples over a 15 min exposure time.

It is worth noting that NO at low concentrations acts as a signalling molecule and, although the NO concentration diffused into the biofilms was not assessed here, it is possible that NO exposure induced bacterial dispersal rather than inactivation [44]. Still, even if NO altered the biofilm structure, its influence on *E*. *coli* biofilms in this work suggests that another plasma-generated agent with antibacterial activity is responsible for the distinct reduction rates between direct and indirect treatment. Heat is another factor that could be responsible for the higher bacterial inactivation in the direct treatment, however previous studies have shown that the gas temperature of the downstream effluent in an air SBD increases by <10°C, putting it close to the optimal growth temperature of *E*. *coli*, suggesting that heating has no substantial effect on the bacterial cells [45].

In contrast to the limited effect of NO on *E*. *coli* biofilms when exposed to plasma, our results indicated UV irradiation can influence the bacterial inactivation kinetics. It is well known that UV efficiently inhibits bacterial replication capability by inducing thymine dimers in DNA strands. There is a debate on whether plasma-emitted UV radiation contributes to the inactivation of microorganisms. Multiple reports indicate the involvement of UV in bacterial kill during plasma exposure [22, 25] and some even suggest that UV photons are the dominant inactivation agent [19]. Contrarily, other studies have concluded that UV has limited or no role in microbial inactivation [23, 24, 26]. Here, plasma-emitted UV increased the bacterial inactivation to the complete kill level. Critically, the experimental setup employed in this study was designed so the synergistic effect of UV and various long-lived reactive species could be examined, an approach not used in previous studies. Based on the results presented in this study, Fig 6, it is plausible to suggest that UV photons are involved in one or more of the following processes: (1) mutagenic effect after interaction with DNA strands, (2) role in photochemical production of oxidants and (3) interaction with biofilm matrix components.

Intracellular DNA fidelity maintenance is critical for the bacterial survival and it has been revealed that exposure to plasma ultimately result in breaks in both single and double strand DNA [46] and mutations [47]. However, it is unlikely that direct interaction with DNA alone is a major mechanism in bacterial inactivation by plasma-emitted UV photons. Previous works in this area have investigated the combined and separate effect of photons and reactive particles generated by a plasma jet, demonstrating that although UV radiation modifies DNA nucleobases, only the particles are capable of causing physical damage to the bacterial cell envelope [46]. Additionally, it has been shown that double-stranded DNA (dsDNA) is largely protected over single-stranded DNA (ssDNA) from plasma photons and particles [48], suggesting that plasma-emitted UV is not sufficient to greatly inactivate bacterial cells but it plays an important role in DNA repair, replication and transcription events where ssDNA is abundant.

In this study, Fig 3A shows the absolute emission intensity from the SBD used for inactivation experiments and there is no quantifiable emission in the UVC portion of the spectrum, thus it is unlikely that the plasma generated UV in this study can directly affect the DNA of *E*. *coli* biofilm cells. Fig 6B confirms this, highlighting the very minor (<0.5 log) reduction in CFU following exposure to plasma-generated UV alone. Consequently, it can be concluded that the discrepancy in the CFU count between UV-treated and -untreated samples in this study is probably due to the secondary effects of UV exposure.

In principal, UV generated from the SBD can interact with the biofilm exopolymeric matrix which serves as an extra defensive barrier to the bacterial cells against environmental stresses such as antibiotics, desiccation, host immune agents and UV itself. Recently, it was reported that chemical and structural changes to the matrix were observed following plasma treatment of *P*. *aeruginosa* [49]. The study identified eDNA as an important protective biofilm agent and the authors suggested that UV photons act on these molecules leading to destabilization of the matrix network, making the biofilm cells more vulnerable to RONS and other reactive agents [49]. Although it has been shown that eDNA is a critical component in the integrity maintenance of *E*. *coli* biofilms [50], the UV alone emitted by the SBD plasma system used in this work accounted for minor biofilm inactivation, ~0.5 log reduction, Fig 6B. Further tests are needed to show whether the UV/eDNA interaction results in matrix changes that would facilitate the penetration of reactive agents into the inner layers of the biofilm.

Perhaps one of the most likely roles of UV in the inactivation process is through the generation of additional radicals directly within the biofilm matrix by photolysis. Plasma generated hydroxyl radicals are mainly produced in the plasma phase and although this radical is a key species in plasma-facing liquids (PFL) systems [32], it has limited penetrating capability into solutions or biological systems, which leads to a very low concentration in aqueous environments. To understand how •OH may be produced deep within a liquid, Park and co-workers [51] used an argon plasma jet to demonstrate that the production of reactive species, including •OH, is enhanced by UV photons via photochemical reactions involving N(III) and H_2_O_2_ as shown in the reactions below. Under UV exposure, H_2_O_2_ and nitrous acid generates ·OH (reactions 1 and 2, respectively) while NO_2_¯ generates ·O^¯^ which is further protonated into ·OH (reactions 3 and 4):

$$H_{2}O_{2}\to \cdot \mathrm{OH}+\cdot \mathrm{OH}$$ (1)

HONO→⋅NO+OH (2)

$${\mathrm{NO}}_{2}^{-}\to \cdot \mathrm{NO}+\cdot O^{-}$$ (3)

$$\cdot O^{-}+H_{2}O\to \cdot OH+\cdot OH^{-}$$ (4)

Furthermore, Schneider and colleagues demonstrated that UV radiation enables the activation of oxygen resulting in the formation of protonated water clusters ions which have been shown to improve the rate of bacterial inactivation [26]. Although the studies mentioned above involved PFLs, water is a major constituent inside and between living cells—and ultimately biofilms—supporting the hypothesis that plasma exposure initiates a series of synergistic interactions between UV photons, the biofilm matrix and the long-lived reactive species.

It has been demonstrated that Gram-negative bacteria are more sensitive than Gram-positive bacteria to CAP, a phenomenon likely to occur due differences in the cell wall thickness [5]. In agreement with our observation, Olatunde and co-workers [52] proposed the production of UV as one of the major factors in the mode of action in Gram-negative bacterial inactivation by CAP. The authors also suggested a reduced relevance of UV transmitted by CAP in the cell envelope damage of Gram-positive bacteria. As this study showed a clear role of UV in *E*. *coli* biofilm inactivation, further studies could elucidate the direct and/or synergistic effect of UV in the cell wall of Gram-positive bacteria.

Ultimately, the findings of this investigation indicate that the long-lived reactive species generated by an SBD in air are primarily responsible for the observed antimicrobial action; however, the efficacy of the approach is significantly enhanced in the presence of plasma generated UV. Given the nature and fluence of the emitted UV it is unlikely that UV photons have a direct impact upon the bacterial cells within the biofilm, or the extracellular components within the biofilm matrix. Consequently, it is surmised that a synergistic interaction between the long-lived chemical species, UV photons and water within the biofilm matrix yield an enhancement in the antimicrobial efficacy of the approach.

## Conclusion

In this contribution a series of experiments have been conducted to unravel the mechanisms of microbial inactivation of *E*. *coli* biofilm exposed to a surface barrier discharge created in atmospheric pressure air. The results indicated that the long-lived plasma generated species are the primary driver behind the observed microbial inactivation; however, the presence of plasma generated UV led to a marked improvement in antimicrobial efficacy. Using both experimental diagnostics and computational modelling it was determined that Nitric Oxide plays a very minor role in the inactivation process and that UV photons emitted by the plasma are unlikely to directly affect bacterial cells within the biofilm matrix. Ultimately, it was postulated that photolysis of water within the biofilm matrix in the presence of long-lived plasma generated species is the most likely mechanism to explain the observed synergistic enhancement in microbial inactivation.

Ultimately, SBD devices are seen as an extremely promising means of bacterial decontamination over large areas. They are attractive not only because of their high antimicrobial efficiency, but because of their broad-spectrum capabilities and their low operating cost, due in part to the consumable free and low energy nature of their operation. This work demonstrates that it is not only the large densities of long-lived reactive chemical agents generated that dictate their efficacy, but the synergy arising from the simultaneous generation of such species and UV photons in the presence of moisture within the microbial sample. Consequently, this work provides a valuable insight into the modes of microbial decontamination mediated by a SBD and can be used to optimise the design of future plasma-based decontamination devices.
